# Antithrombotic Therapy in Primary and Secondary Prevention of Coronary Artery Disease

**DOI:** 10.3390/jcm15114248

**Published:** 2026-05-30

**Authors:** Giacinto Di Leo, Marco Spagnolo, Daniele Giacoppo, Antonio Greco, Davide Capodanno

**Affiliations:** Division of Cardiology, A.O.U. Policlinico “G. Rodolico—San Marco”, University of Catania, 95123 Catania, Italy; giacinto.dileo@studium.unict.it (G.D.L.); davide.capodanno@unict.it (D.C.)

**Keywords:** coronary artery disease, antithrombotic therapy, dual antiplatelet therapy, P2Y_12_ inhibitors, bleeding risk

## Abstract

Antithrombotic therapy is central to the management of coronary artery disease (CAD), yet its optimal use requires a continuous balance between ischemic protection and bleeding risk. While aspirin has historically been the cornerstone of treatment, contemporary evidence supports a transition toward increasingly individualized strategies across the spectrum of disease. In primary prevention, the role of aspirin remains marginal and is limited to carefully selected high-risk individuals. Following percutaneous coronary intervention (PCI), dual antiplatelet therapy (DAPT) remains the standard of care; however, both its duration and composition are progressively tailored according to patient-specific ischemic and bleeding risk profiles. In chronic coronary syndromes, shorter DAPT followed by single antiplatelet therapy—particularly P2Y_12_ inhibitor monotherapy—has emerged as an effective bleeding-avoidance strategy without compromising ischemic outcomes. In acute coronary syndromes, 12 months of DAPT remains the recommended approach, although de-escalation strategies may be considered in selected patients at lower ischemic risk. For long-term secondary prevention, emerging evidence suggests a potential advantage of clopidogrel over aspirin, while in patients with persistently high ischemic risk, intensified antithrombotic regimens may provide additional benefit. Special populations require tailored treatment strategies. Overall, contemporary evidence supports a paradigm shift toward a precision medicine approach in CAD, in which antithrombotic therapy is dynamically adapted to the individual balance between ischemic and bleeding risk to optimize long-term clinical outcomes.

## 1. Introduction

Coronary artery disease (CAD) is a pathological condition characterized by the accumulation of atherosclerotic plaque within the epicardial coronary arteries. Its dynamic nature underlies the broad clinical spectrum of ischemic heart disease, ranging from chronic coronary syndromes (CCS) to acute coronary syndromes (ACS), including unstable angina, non-ST-segment elevation myocardial infarction (NSTEMI), and ST-segment elevation myocardial infarction (STEMI) [1]. Despite substantial advances in prevention and risk factor control, CAD remains a leading cause of morbidity and mortality worldwide [2]. According to the American Heart Association report on US and global data published in 2026, in 2021 CAD affected approximately 254 million individuals globally and was responsible for about 8.99 million deaths [3].

Because coronary thrombosis plays a central role in the pathophysiology of CAD and its clinical complications, antithrombotic therapy has become a cornerstone of both prevention and treatment across the continuum of disease [4]. In primary prevention, the objective is to reduce the risk of first ischemic events in individuals without overt CAD, although this strategy remains controversial because the modest reduction in ischemic events may be offset by an increased risk of bleeding [4]. In contrast, in secondary and tertiary prevention—aimed, respectively, at reducing the risk of recurrent cardiovascular events and mitigating the long-term burden of CAD by limiting complications, preserving functional capacity, and improving quality of life—antithrombotic therapy provides substantial protection against recurrent ischemic events and remains a fundamental component of long-term management [5,6].

Over the past three decades, the antithrombotic armamentarium has expanded considerably, moving from aspirin-based regimens to more complex and individualized strategies that include dual antiplatelet therapy (DAPT), P2Y_12_ inhibitor monotherapy, prolonged DAPT in selected patients, and dual-pathway inhibition with combined antiplatelet and anticoagulant agents [7,8]. At the same time, advances in stent technology, improved understanding of the temporal distribution of ischemic and bleeding risk, and the increasing recognition of patient heterogeneity have progressively shifted clinical practice away from a “one-size-fits-all” approach toward more personalized treatment algorithms [9,10,11].

This review provides a contemporary overview of antithrombotic therapy in CAD, covering primary prevention, antithrombotic strategies in ACS and CCS, long-term secondary prevention, and management in selected special populations. It summarizes the most recent randomized clinical trials investigating abbreviated DAPT regimens, P2Y_12_ inhibitor monotherapy, aspirin withdrawal strategies, and de-escalation approaches (Supplementary Material), including emerging strategies that were not consistently addressed in earlier reviews. Particular emphasis is placed on strategies for tailoring antiplatelet therapy according to the clinical setting, with careful consideration of the individual patient’s ischemic and bleeding risk.

## 2. Rationale for Antithrombotic Therapy in Coronary Artery Disease

CAD is a dynamic atherosclerotic disease characterized by the progressive development, remodeling, and sometimes destabilization of coronary plaques [12]. Its clinical manifestations may arise either from acute plaque disruption, with subsequent atherothrombosis leading to spontaneous myocardial infarction (e.g., type 1), or from gradual plaque progression and spontaneous plaque healing, which can increase plaque burden and produce severe flow-limiting stenoses even in the absence of overt thrombosis (e.g., type 2 myocardial infarction) [13]. These pathophysiological mechanisms underlie the clinical conditions of NSTEMI, which typically results from a partially occluded coronary artery leading to subendocardial ischemia, and STEMI, which is usually caused by complete vessel occlusion, resulting in transmural myocardial ischemia [1,14]. Consequently, antithrombotic therapy is the cornerstone of treatment and secondary prevention of myocardial infarction [1,8].

Following a myocardial infarction diagnosis, patients are typically loaded with aspirin and initiated on heparin to halt clot progression and facilitate thrombus dissolution [1]. During coronary angiography and percutaneous coronary intervention (PCI), heparin therapy is continued to prevent catheter-related thrombosis, and patients receive a loading dose of a P2Y_12_ inhibitor (e.g., oral clopidogrel, ticagrelor, or prasugrel, or intravenous cangrelor) [1,15,16]. Subsequently, DAPT—a combination of aspirin and a P2Y_12_ inhibitor—is maintained for a variable duration ranging from months to a year, tailored to the patient’s thrombotic and bleeding risk, to prevent stent thrombosis and new ischemic events from non-culprit lesions [1,17]. In the long term (e.g., secondary and tertiary prevention), most patients transition to lifelong single antiplatelet therapy in the prevention of recurrent plaque or stent thrombosis, which is well-established in this setting, unlike primary prevention, where evidence and recommendations remain uncertain [7,18,19,20].

## 3. Antithrombotic Therapy for Primary Prevention

While evidence supports the use of antiplatelet therapy for the secondary and tertiary prevention of coronary ischemic events in patients with established atherosclerosis, prior myocardial infarction, or previous myocardial revascularization, demonstrating that its benefits outweigh the associated bleeding risk, the role of antiplatelet therapy in the primary prevention of ischemic events among individuals without overt CAD remains controversial [21,22,23].

The evidence supporting the antiplatelet strategy for primary prevention almost completely relies on aspirin monotherapy [24]. In a Bayesian meta-analysis of 13 trials including 164,225 participants with 1,050,511 person-years of follow-up, aspirin treatment was associated with a significant reduction in the composite of cardiovascular death, nonfatal myocardial infarction, or nonfatal stroke compared with no aspirin treatment (60.2 per 10,000 person-years versus 65.2 per 10,000 person-years; hazard ratio [HR] 0.89, 95% credible interval [CrI] 0.84–0.94) [4]. These findings corresponded to an absolute risk reduction of 0.41% (95% CrI 0.23–0.59) and a number needed to treat to benefit of 241, reflecting the modest magnitude of the reduction in ischemic events and the large number of treated patients required to prevent a single event [4]. However, aspirin treatment was also associated with an increased risk of major bleeding events compared with no aspirin treatment (23.1 per 10,000 person-years versus 16.4 per 10,000 person-years; HR 1.43, 95% CrI 1.30–1.56) [4]. These findings corresponded to an absolute risk increase of 0.47% (95% CrI 0.34–0.62%) and a number needed to harm of 210, indicating that, in a general mixed population, the net clinical benefit of aspirin monotherapy for primary prevention is marginal compared to no treatment [4].

The most recent large-scale randomized trial on aspirin for primary prevention focused on specific higher-risk clinical settings. Nevertheless, their results are heterogeneous and it remains unclear whether a personalized approach to identify individuals without evident cardiovascular disease who may derive benefit from aspirin mono-therapy is applicable [25,26,27]. Specifically, ASCEND, ARRIVE, and ASPREE enrolled patients with diabetes, subjects with multiple cardiovascular risk factors without diabetes, and adults aged 65–70 years or older, respectively [25,26,27]. At a mean follow-up of 7.4 years, the ASCEND trial demonstrated a significant reduction in vascular death, myocardial infarction, or ischemic cerebrovascular events in patients assigned to aspirin compared with those assigned to placebo (8.5% vs. 9.6%; rate ratio [RR] 0.88, 95% confidence interval [CI] 0.79–0.97) [27]. However, in the same trial, the risk of major bleeding was significantly higher in patients assigned to aspirin compared with those assigned to placebo (4.1% vs. 3.2%; RR 1.29, 95% CI 1.09–1.52) [27]. At a median follow-up of 5 years, in the ARRIVE trial, aspirin was not associated with a significant reduction in major cardiovascular ischemic events compared with placebo (4.29% versus 4.48%; hazard ratio [HR] 0.96, 95% CI 0.81–1.13) [25]. Similarly, at a median follow-up of 4.7 years, in the ASPREE trial, major cardiovascular ischemic events were not significantly different between treatment groups (10.7 events per 1000 person-years versus 11.3 events per 1000 person-years; HR 0.95, 95% CI 0.83–1.08) [26]. Of note, in the ASPREE trial, major bleeding was significantly higher in the aspirin group compared with the placebo group (8.6 events per 1000 person-years versus 6.2 events per 1000 person-years; HR 1.38, 95% confidence interval 1.18–1.62) [26]. In the ARRIVE trial, gastrointestinal bleeding occurred more frequently in the aspirin group compared with the placebo group (0.97% vs. 0.46%; HR 2.11, 95% CI 1.36–3.28) [25].

## 4. Antithrombotic Therapy for Secondary Prevention

### 4.1. Antiplatelet Therapy in Chronic Coronary Syndromes After Percutaneous Coronary Intervention

Oral antiplatelet therapy is a central component of both short- and long-term management of patients with CCS undergoing PCI [8]. With the advent of PCI, DAPT rapidly became the standard of care in patients with CCS undergoing coronary revascularization, representing a cornerstone for the prevention of periprocedural and postprocedural ischemic events [28,29,30]. In the era of first-generation drug-eluting stents (DES), a minimum DAPT duration of 12 months was empirically recommended regardless of clinical presentation [31]. However, after PCI, thrombotic risk is highest during the first month—particularly within the first two weeks—and declines progressively over the subsequent year, whereas bleeding risk peaks in the periprocedural period and thereafter remains relatively constant [32]. This temporal dissociation between ischemic and haemorrhagic risk, together with substantial improvements in DES technology and PCI techniques, provided the biological rationale for exploring shorter durations of DAPT and for limiting prolonged exposure to DAPT beyond the early postprocedural phase [11,32,33].

**Short DAPT Followed by Aspirin Monotherapy**—Early randomized trials comparing 6 versus 12–24 months of DAPT consistently showed no ischemic benefit with prolonged therapy but an increased risk of bleeding (Table 1). Studies such as EXCELLENT, PRODIGY, SECURITY, ITALIC, ISAR-SAFE, I-LOVE-IT 2, OPTIMA-C, and NIPPON demonstrated the noninferiority of 6-month DAPT compared with longer regimens, although several trials were limited by early termination or low event rates [34,35,36,37,38,39,40,41]. Shorter durations of DAPT were further evaluated in the RESET and OPTIMIZE trials, which tested 3 months of DAPT and did not show an increase in ischemic events; however, these findings were limited by the use of a single stent platform [42,43]. A subsequent meta-analysis confirmed that DAPT durations of 6 months or less were not associated with higher rates of mortality, myocardial infarction, or stent thrombosis compared with 12 months, while significantly reducing major bleeding [44]. More recent trials have explored ultra-short DAPT strategies of 1 month. In the One-Month DAPT trial, 1-month DAPT followed by aspirin after polymer-free drug-coated stent implantation was noninferior to 6–12 months of DAPT with biodegradable-polymer DES for a composite endpoint of cardiovascular events and major bleeding, although interpretation was limited by different stent platforms [45]. Finally, The MASTER DAPT trial provided more definitive evidence on early aspirin discontinuation in a high-bleeding-risk population. Among event-free patients at 1 month after PCI, an abbreviated DAPT followed by aspirin or clopidogrel was noninferior to standard DAPT with respect to net adverse clinical events and major adverse cardiovascular events (MACE), while significantly reducing clinically relevant bleeding. These results support a 1-month DAPT strategy in selected high-bleeding-risk patients treated with contemporary DES [46].

**Short DAPT Followed by P2Y_12_ Receptor Inhibitor Monotherapy**—In three randomized trials—SMART-CHOICE, TWILIGHT, and SHARE—a strategy of 3 months of DAPT followed by P2Y_12_ inhibitor monotherapy was noninferior to 12 months of DAPT with respect to ischemic outcomes, while consistently reducing bleeding [47,48,49]. However, important differences in study design and enrolled populations should be considered when interpreting these findings. TWILIGHT was designed as a superiority trial and predominantly enrolled Caucasian patients with at least one clinical and one angiographic feature associated with increased ischemic or bleeding risk, with ticagrelor used as the exclusive P2Y_12_ inhibitor [49]. In contrast, SMART-CHOICE and SHARE were non-inferiority trials conducted exclusively in East Asian populations, in which clopidogrel was the most frequently prescribed P2Y_12_ inhibitor. These differences may affect the generalizability of the results to broader patient populations and routine clinical practice [47,48].

Trials evaluating a 1-month DAPT strategy yielded more heterogeneous results. In STOPDAPT-2, 1 month of DAPT followed by clopidogrel monotherapy was both noninferior and superior to 12 months of DAPT for a composite of cardiovascular and bleeding events, largely driven by a reduction in bleeding. Although the wide noninferiority margin and predominantly East Asian population warrant cautious interpretation [50]. By contrast, GLOBAL LEADERS failed to demonstrate superiority of ticagrelor monotherapy after 1-month DAPT compared with standard DAPT followed by aspirin (Table 2) [51]. However, the adjudicated GLASSY substudy suggested noninferiority of ticagrelor monotherapy to 12-month DAPT in terms of ischemic events, with similar rates of major bleeding [52]. An individual patient-level meta-analysis including six randomized trials and 25,960 patients undergoing PCI assessed whether ticagrelor or clopidogrel monotherapy after short-course DAPT was noninferior to standard DAPT with respect to death, myocardial infarction, or stroke. Ticagrelor monotherapy met the criterion for noninferiority and was associated with significantly lower rates of major bleeding and net adverse clinical events. Clopidogrel monotherapy similarly reduced major bleeding but did not meet noninferiority criteria for ischemic outcomes [53]. Collectively, these trials support early aspirin withdrawal in selected patients, while underscoring the importance of treatment duration, choice of P2Y_12_ inhibitor, and patient risk profile in interpreting outcomes. More recently, a patient-level meta-analysis showed that P2Y_12_ inhibitor monotherapy—with either clopidogrel or ticagrelor—may be preferred over aspirin monotherapy after DAPT discontinuation for secondary prevention in patients undergoing PCI, regardless of clinical presentation, due to greater ischemic protection without a concurrent increase in bleeding risk [22].

**Figure 1:**
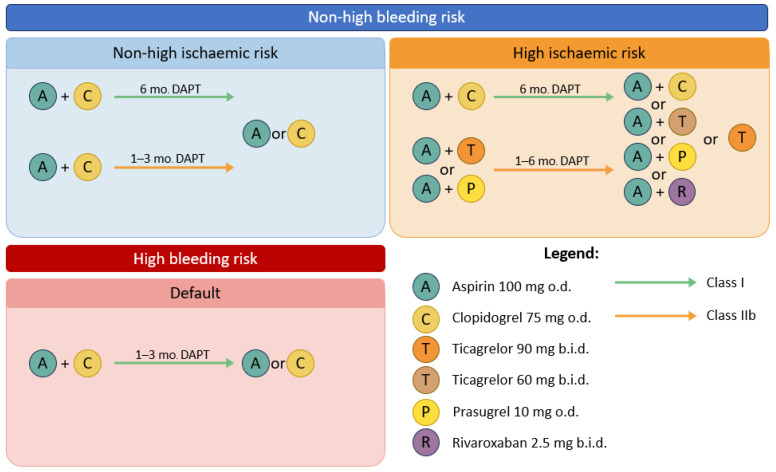
Antithrombotic therapy algorithm in chronic coronary syndrome patients.

**Supporting Guidelines**—Post-PCI antithrombotic management in CCS demonstrates substantial concordance between American and European guidelines (Figure 1). Both guidelines recommend DAPT with aspirin and clopidogrel for up to 6 months as the default strategy, followed by SAPT (Class I, Level A). In patients at high bleeding risk but not at high ischemic risk, early discontinuation of DAPT after 1 to 3 months with continuation of monotherapy is recommended (Class I, Level A); a similar shortening of DAPT duration may also be considered in patients at intermediate ischemic risk (Class IIb, Level B). In this setting, American guidelines further support P2Y_12_ inhibitor monotherapy after 1 to 3 months of DAPT in selected patients treated with DES as a bleeding-avoidance strategy (Class IIa, Level A) [19,54].

Patients are categorized according to bleeding risk (non-high vs. high) and ischemic risk (non-high vs. high). Arrows indicate the recommended duration of DAPT, and their colors represent the classes of recommendation (I, IIa, IIb) in accordance with current European Society of Cardiology (ESC) guidelines. In patients with non-high bleeding risk and non-high ischaemic risk, the strategy includes 6 months of DAPT (Aspirin + Clopidogrel; Class I) or a shorter 1–3 month DAPT course (Class IIa) followed by monotherapy. In the high ischaemic risk setting, 6 months of DAPT with Clopidogrel (Class I) or 1–6 months with potent P2Y_12_ inhibitors (Class IIb) are illustrated, followed by extended dual therapy options (Class IIa) or Ticagrelor monotherapy (Class IIb). For patients at high bleeding risk, the default strategy consists of a shortened 1–3 month DAPT duration with Aspirin and Clopidogrel (Class I) followed by single antiplatelet therapy.

**Ongoing trials**—Ongoing randomized trials are actively exploring multiple strategies for modulation of DAPT in patients with CCS undergoing PCI (Table 3). The TAILOR-DAPT trial (NCT05732701) is investigating PRECISE-DAPT–guided personalization of DAPT duration, while OPTIMIZE-APT (NCT05418556), ANGIODAPT (NCT05952206) and SHORTDAPT (NCT06648720) are assessing aspirin discontinuation after 1 month (or after 3 months in ACS patients enrolled in the OPTIMIZE-APT trial), with transition to P2Y_12_ inhibitor monotherapy. Similarly, GENOSS-DAPT (NCT05770674) compares 1-month DAPT followed by clopidogrel monotherapy with standard 12-month DAPT. Finally, additional trials are exploring aspirin-free strategies early after PCI. The TICALONE trial (NCT06509893) and the PROMOTE trial (NCT06916520) are comparing P2Y_12_ inhibitor monotherapy—ticagrelor and reduced-dose prasugrel, respectively—with standard DAPT in patients undergoing PCI.

### 4.2. Antiplatelet Therapy in Acute Coronary Syndromes After Percutaneous Coronary Intervention

At the turn of the millennium, antithrombotic therapy for ACS was largely limited to aspirin, and the clinical role of P2Y_12_ receptor inhibitors remained incompletely defined. Over the subsequent two decades, a series of randomized trials established DAPT as a cornerstone of treatment, particularly in patients undergoing PCI. The CURE trial first demonstrated that adding clopidogrel to aspirin significantly reduced ischemic events among patients with unstable angina or NSTEMI, albeit at the cost of increased bleeding [55]. Subsequent large trials—TRITON-TIMI 38 and PLATO—demonstrated the superiority of newer oral potent P2Y_12_ inhibitors, prasugrel and ticagrelor, over clopidogrel, again with a trade-off of higher bleeding rates [56,57]. Comparative efficacy between prasugrel and ticagrelor has been less clear: while PRAGUE-18 found no significant differences, ISAR-REACT 5 reported lower rates of MACE with prasugrel without increased bleeding [58,59,60,61]. However, a subsequent network meta-analysis incorporating direct and indirect comparisons across oral P2Y_12_ inhibitors in ACS found no significant difference between prasugrel and ticagrelor; relative to clopidogrel, prasugrel was associated with a reduction in myocardial infarction, whereas ticagrelor reduced all-cause and cardiovascular mortality [62]. More recently, the TUXEDO-2 trial failed to demonstrate the non-inferiority of ticagrelor compared with prasugrel in diabetic patients with multivessel coronary disease undergoing PCI [63]. Taken together, these data supported the empirical adoption of 12 months of DAPT after ACS, despite persistent uncertainty about the optimal balance between ischemic benefit and bleeding risk [64,65].

**Short DAPT Followed by Aspirin Monotherapy**—Advances in stent technology and a clearer understanding of the differing temporal distributions of ischemic and bleeding risk after PCI have since prompted strategies that modulate both the intensity and duration of platelet inhibition [32]. Early efforts focused on shortening the overall duration of DAPT by early discontinuation of the P2Y_12_ inhibitor (Table 4). In this regard, DAPT-STEMI showed noninferiority of 6 versus 12 months of DAPT in patients with STEMI; however, randomization was restricted to patients who remained event-free during the first 6 months after PCI, thereby selecting a lower-risk population. The low overall event rate and the wide noninferiority margin further limit the interpretability of the findings [66]. In contrast, SMART-DATE—although meeting its noninferiority criterion for the composite endpoint—reported an excess of myocardial infarction with abbreviated DAPT [67]. Similarly, REDUCE demonstrated higher rates of mortality and stent thrombosis with a 3-month DAPT regimen [68]. Notwithstanding methodological limitations in some trials, these findings suggest caution regarding premature cessation of P2Y_12_ inhibition at 3–6 months and support such approach primarily in patients at high bleeding risk.

**Short DAPT Followed by P2Y_12_ Monotherapy**—Concurrently, attention has turned to “aspirin-free” strategies that withdraw aspirin early and continue monotherapy with a P2Y_12_ inhibitor (Table 5), an approach supported by evolving insights into platelet biology [69,70,71]. TICO demonstrated noninferiority of ticagrelor monotherapy after 3 months of DAPT, with a benefit driven principally by reductions in bleeding [72]. By contrast, in STOPDAPT-2 ACS, a 1-month DAPT course followed by clopidogrel monotherapy was associated with increased ischemic events [73]. More recently, trials including T-PASS, ULTIMATE-DAPT, and 4D-ACS have suggested that aspirin discontinuation after one month-DAPT, followed by potent P2Y_12_ monotherapy, can preserve ischemic protection comparable to conventional DAPT while substantially reducing bleeding [74,75,76]. However, relevant differences in trial design and treatment strategy should be considered. In T-PASS trial, an open-label, multicenter trial, aspirin was discontinued at a median of 16 days after PCI, followed by ticagrelor monotherapy [76]. This strategy was associated with a reduction in net clinical events compared with standard DAPT, driven primarily by a marked decrease in major bleeding (BARC 3–5), without an apparent increase in ischemic events [76]. Nevertheless, the overall low event rates suggest the enrollment of a relatively low-risk population, which should be considered when interpreting these findings. By contrast, ULTIMATE-DAPT adopted a more rigorous methodological design, being randomized, placebo-controlled, and double-blind [74]. Aspirin was systematically discontinued 1 month after PCI, and ticagrelor monotherapy was superior to standard DAPT in reducing clinically relevant bleeding (BARC type 2, 3, or 5), while preserving noninferiority with respect to MACCE [74]. In 4D-ACS, patients were assigned after 1 month of standard DAPT to either prasugrel monotherapy (5 mg daily) or continued DAPT with prasugrel (5 mg) plus aspirin. was both noninferior and superior with respect to net adverse clinical events, primarily owing to a reduction in clinically relevant bleeding (BARC type 2, 3, or 5), whereas ischemic event rates were similar between groups [75]. Although the findings of these trials were directionally consistent, differences in trial design, bleeding definitions, and antiplatelet regimens should be considered in their interpretation. In addition, the predominance of Asian populations may limit generalizability to broader clinical settings.

More recently, even shorter aspirin exposure has been investigated. STOPDAPT-3 highlighted the limitations of immediate “aspirin-free” strategies, reporting increases in unplanned revascularization and subacute stent thrombosis [77]. The most recent trials—NEO-MINDSET and TARGET-FIRST—have further emphasized that the timing of aspirin withdrawal, patient selection, and careful stratification of ischemic and bleeding risk are critical determinants of the safety and efficacy of aspirin-free approaches. NEO-MINDSET showed that aspirin withdrawal at the time of PCI failed to preserve ischemic risk equivalence to standard DAPT despite reducing bleeding, with a numerical increase in ischemic endpoints—including stent thrombosis—predominantly in the subacute period [78]. A prespecified analysis revealed important heterogeneity according to clinical presentation: early aspirin discontinuation was not associated with excess ischemic risk among patients with NSTEMI but was accompanied by a significant increase in 1-year ischemic events among patients with STEMI [79]. By contrast, TARGET-FIRST reported that, in carefully selected patients at low ischemic risk with complete revascularization, aspirin discontinuation after at least one month of DAPT was noninferior to conventional therapy and was associated with a significant reduction in clinically relevant bleeding [80]. These observations underscore that ischemic and bleeding risk profiles—closely tied to clinical presentation and patient characteristics—substantially influence the safety of abbreviated DAPT strategies [81]. In summary, contemporary evidence suggests that abbreviated DAPT strategies—particularly those involving aspirin discontinuation after at least 1 month—may be safe in selected patients with ACS, as they have been shown to be noninferior to standard-duration DAPT while reducing bleeding risk. By contrast, aspirin discontinuation immediately after PCI or within the first 7 days appears concerning, as it has been associated with a substantially increased risk of ischemic events, likely owing to inadequate antithrombotic protection during the period of greatest thrombotic vulnerability. Accordingly, individualized antithrombotic therapy, guided by clinical presentation, anatomic complexity, and careful assessment of ischemic and bleeding risks, remains central to the management of ACS. Recent meta-analyses showed that, in ACS patients undergoing PCI, P2Y_12_ inhibitor monotherapy after short DAPT significantly reduced net adverse clinical events, as well as any bleeding and major bleeding, whereas aspirin monotherapy yielded neutral results [17,22,82,83].

**Figure 2:**
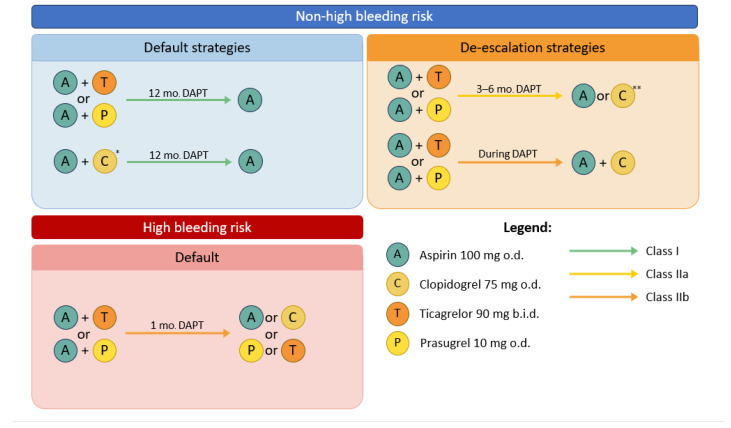
Antithrombotic therapy algorithm in acute coronary syndrome patients. * Clopidogrel is recommended when prasugrel or ticagrelor are not available, cannot be tolerated, or are contraindicated (Level of evidence C). ** Preferably with a P2Y12 receptor inhibitor.

**Supporting Guidelines**—Contemporary guidelines provide a structured approach to antithrombotic therapy in patients presenting with ACS, balancing the need for potent ischemic protection during the acute and subacute phases against the risk of bleeding, with treatment duration and intensity tailored to individual patient risk profiles (Figure 2). Both guidelines generally endorse 12 months of DAPT, preferably with prasugrel or ticagrelor (Class I, Level B), reserving clopidogrel (Class I, Level C) for patients with contraindications or intolerance to more potent P2Y_12_ inhibitors [1,84]. To mitigate bleeding risk, both guidelines endorse de-escalation strategies, defined as a reduction in the intensity of platelet inhibition through modification of the antiplatelet agent, dose, or number of drugs used [1,84,85]. European guidelines allow discontinuation of one antiplatelet agent after 3 to 6 months in patients at low thrombotic risk (Class IIa, Level A) or after 1 month in patients at high bleeding risk (Class IIb, Level B). American guidelines permit shortening of DAPT to 1 month in patients at high bleeding risk, with discontinuation of either aspirin or the P2Y_12_ inhibitor (Class IIb), and recommend ticagrelor monotherapy after 1 month of DAPT as a strategy to reduce bleeding risk (Class I). In addition, both guidelines suggest that switching from ticagrelor or prasugrel to clopidogrel after 1 month may be considered in patients in whom bleeding risk predominates (Class IIb) [1,84].

Patients are stratified according to bleeding risk. For each risk category, default and de-escalation strategies of dual antiplatelet therapy (DAPT) are illustrated. Arrows indicate the recommended duration of DAPT and their color represents the classes of recommendation (I, IIa, IIb) in accordance with current European Society of Cardiology (ESC) guidelines. In the non-high bleeding risk setting, the default strategy consists of 12 months of DAPT using Aspirin plus a potent P2Y_12_ inhibitor (Ticagrelor or Prasugrel) or Clopidogrel as an alternative. De-escalation strategies for these patients include shortening DAPT duration to 3–6 months followed by monotherapy (Class IIa) or switching from a potent P2Y_12_ inhibitor to Clopidogrel during the DAPT course (Class IIb). In patients at high bleeding risk, the default strategy involves a shortened 1-month DAPT duration followed by single antiplatelet therapy (Class IIb).

**Ongoing randomized trials**—Several ongoing randomized trials are expected to further refine the minimal safe duration and optimal composition of antiplatelet therapy in ACS (Table 6). The PREMIUM trial (NCT05709626) and the STARS-DAPT trial (NCT05785897) are evaluating prasugrel or ticagrelor monotherapy compared with conventional DAPT in patients with STEMI. The COMPARE STEMI-ONE trial (NCT05491200) evaluates a short-course DAPT (i.e., 30 to 45 days) strategy followed by prasugrel monotherapy versus conventional DAPT, with net adverse clinical events (NACE) at 11 months as the primary outcome. SORT OUT XII DAPT Duration Trial (NCT06718179) randomizes patients with ACS to 1 month of DAPT followed by prasugrel monotherapy versus 12 months of DAPT, with 1-year any bleeding and a composite ischemic as co-primary endpoints. Alternative de-escalation strategies are being explored in TIGER (NCT04255602), which reduces ticagrelor maintenance dose from 90 mg to 60 mg twice daily after the first month of standard therapy while continuing aspirin and in MATE (NCT04937699), which evaluates a hybrid regimen combining DAPT, ticagrelor monotherapy, and subsequent clopidogrel monotherapy. Finally, ELECTRA-SIRIO 2 (NCT04718025) is comparing low-dose ticagrelor with or without aspirin to standard-dose ticagrelor-based DAPT.

## 5. Long-Term Antithrombotic Therapy

For long-term secondary prevention, aspirin has traditionally been the cornerstone of antithrombotic monotherapy, as multiple studies have demonstrated that its benefits in reducing the risk of myocardial infarction and ischemic stroke outweigh the associated risk of bleeding [21]. However, evidence supporting monotherapy with a P2Y_12_ inhibitor has progressively accumulated (Table 7). Evidence derives from the CAPRIE trial and was further strengthened by subsequent studies such as HOST-EXAM and SMART-CHOICE 3, which overall suggest that clopidogrel may reduce ischemic events compared to ASA [86,87,88,89]. Notably, the 10-year follow-up of HOST-EXAM showed that clopidogrel monotherapy was associated with a lower incidence of the composite endpoint, as well as reduced thrombotic and bleeding events compared with aspirin, with no difference in all-cause mortality [90]. More recently, a patient-level meta-analysis demonstrated that long-term clopidogrel therapy was superior to aspirin monotherapy in preventing major adverse cardiovascular and cerebrovascular events, without an increase in bleeding risk [23]. These findings were further expanded by another individual patient-level meta-analysis, which compared P2Y_12_ inhibitor monotherapy—either clopidogrel or ticagrelor—with aspirin monotherapy for long-term secondary prevention. P2Y_12_ inhibitor monotherapy was associated with a lower risk of cardiovascular death, myocardial infarction, and stroke, primarily driven by a reduction in myocardial infarction, while major bleeding rates were similar between treatment strategies. In addition, net adverse clinical events were significantly lower with P2Y_12_ inhibitor monotherapy, with consistent treatment effects across prespecified subgroups and according to the type of P2Y_12_ inhibitor used [91]. Collectively, these findings challenge the historical role of aspirin as the default long-term antiplatelet strategy and support P2Y_12_ inhibitor monotherapy as a potential alternative for secondary prevention in patients with established CAD.

Among patients with CCS who remain at high ischemic risk and do not have a high bleeding risk, several strategies of treatment intensification have been evaluated. Early evidence from the CHARISMA trial did not show a clear benefit of DAPT with aspirin and clopidogrel in a broad population with atherosclerotic disease, although subgroup analyses suggested benefit in patients with prior ischemic events [92,93]. Trials evaluating extended DAPT after PCI have yielded heterogeneous results. Pooled analyses of the REAL-LATE and ZEST-LATE trials—collectively referred to as DES-LATE—as well as the OPTIDUAL trial, yielded neutral results. In contrast, the DAPT trial demonstrated a significant reduction in stent thrombosis and myocardial infarction, at the expense of an increased risk of bleeding, with the net clinical benefit largely confined to patients with a prior myocardial infarction [94,95,96,97].

Evidence regarding long-term DAPT with aspirin plus ticagrelor is mainly derived from the PEGASUS–TIMI 54 trial, which showed that ticagrelor added to aspirin in patients with prior myocardial infarction reduced major adverse cardiovascular events but increased major bleeding, with the 60 mg twice-daily dose providing a more favorable efficacy–safety balance [98]. Similarly, the THEMIS trial evaluated ticagrelor plus aspirin in patients with stable coronary artery disease and diabetes, showing a reduction in ischemic events at the expense of increased major bleeding [99]. Finally, the COMPASS trial demonstrated that dual-pathway inhibition with aspirin plus very-low-dose rivaroxaban reduced major ischemic events and mortality compared with aspirin alone, with an increase in major bleeding but no excess in fatal or intracranial hemorrhage [100]. Overall, prolonged or intensified antithrombotic therapy may provide additional ischemic protection in carefully selected CCS patients, and treatment decisions should be individualized according to the balance between ischemic and bleeding risk and the eligibility criteria of the pivotal trials supporting these strategies.

Contemporary guidelines provide a framework for short and long-term antithrombotic therapy in patients with CCS, integrating individual ischemic and bleeding risk to guide the duration and intensity of treatment (Figure 1). In patients with CCS, the European and American guidelines largely agree on a long-term strategy centered on SAPT, individualized according to ischemic and bleeding risk. Both recommend lifelong low-dose aspirin (75–100 mg daily) after an initial period of DAPT in patients with prior myocardial infarction or remote PCI, with clopidogrel as an effective alternative in those with aspirin intolerance (Class I, Level A). American guidelines specifically discourage the routine addition of clopidogrel to aspirin in stable patients without recent ACS or PCI (Class III), whereas European guidelines allow consideration of adding a second antithrombotic agent in selected patients at high ischemic and low bleeding risk (Class IIa, Level A) [19,54]. Conversely, in patients with prior myocardial infarction who remain at high ischemic risk but without excessive bleeding risk, American guidelines allow the extension of DAPT beyond 12 months, for up to 3 years, to reduce MACE (Class IIb, Level A). In the context of high thrombotic-risk stenting, European guidelines permit short-term DAPT with prasugrel or ticagrelor for 3 to 6 months (Class IIb, Level C), and consider ticagrelor monotherapy (90 mg twice daily) as an alternative to prolonged DAPT (Class IIb, Level C). Finally, in patients with high ischemic and low-to-moderate bleeding risk, American guidelines consider the addition of low-dose rivaroxaban (2.5 mg twice daily) to aspirin reasonable for long-term reduction in MACE (Class IIa, Level B) [19,54].

Several ongoing trials (Table 8) are currently investigating optimal long-term antiplatelet strategies following short-term DAPT. The ongoing C-MODE trial (NCT05320926) directly compares clopidogrel versus aspirin monotherapy following 1–3 months of DAPT in patients with CCS or non–myocardial infarction ACS, with stratification according to bleeding risk, clinical presentation, and lesion complexity. Other studies are evaluating the role of long-term SAPT versus prolonged DAPT. The A-CLOSE trial (NCT03947229) randomizes event-free patients at high ischemic or bleeding risk to clopidogrel monotherapy or extended DAPT beyond 12 months after DES implantation. In contrast, the DAPT-MVD trial (NCT04624854) focuses on patients with coronary multivessel disease, comparing extended DAPT with aspirin plus clopidogrel versus aspirin monotherapy beyond 12 months.

## 6. Antithrombotic Therapy in Special Populations

### 6.1. Antithrombotic Therapy After Coronary Artery Bypass Grafting

Chronic Coronary Syndrome—In the context of CABG, available evidence indicates that aspirin monotherapy remains an effective and safe strategy for preserving graft patency [101,102,103,104]. The evidence on clopidogrel monotherapy remains inconsistent, with a CAPRIE subgroup analysis suggesting a benefit over aspirin in patients with prior CABG, whereas subsequent substudies have not consistently demonstrated a clear clinical advantage [105,106,107]. The potential role of ticagrelor monotherapy after CABG has been evaluated in the TiCAB and TARGET trials, which compared ticagrelor with aspirin, without demonstrating statistically significant differences in MACE and graft occlusion, respectively [108,109]. In contrast, studies evaluating DAPT after CABG have yielded heterogeneous results and have generally been limited by small sample sizes. In DACAB study, ticagrelor plus aspirin reduced saphenous vein graft failure compared with aspirin alone, and extended follow-up suggested a lower incidence of MACE with DAPT [110,111]. A network meta-analysis including 20 randomized trials showed that both ticagrelor-based DAPT and clopidogrel-based DAPT were associated with a significantly lower risk of saphenous vein graft failure than aspirin monotherapy [112]. On the basis of the available evidence, DAPT with either ticagrelor or clopidogrel for up to 12 months may be considered in patients with CCS who are not at high bleeding risk, particularly when prevention of graft failure is a primary therapeutic goal [104]. The role of dual-pathway inhibition after CABG remains uncertain. In the COMPASS-CABG substudy rivaroxaban plus aspirin showed a nonsignificant reduction in MACE—consistent with the main trial—but did not reduce 1-year graft failure, suggesting no clear benefit of routine anticoagulation after CABG [113].

Acute Coronary Syndrome—Evidence supporting the use of DAPT after CABG in patients with ACS is derived largely from subgroup analyses of major ACS trials, including CURE, PLATO, and TRITON–TIMI 38, which showed treatment effects consistent with those observed in the overall study populations [114,115,116]. However, interpretation of these data is limited by randomization at index presentation, incomplete resumption of P2Y_12_ inhibition after surgery, and the underrepresentation of patients at high bleeding risk [104]. A meta-analysis of 38 studies found that DAPT, compared with single antiplatelet therapy, reduced all-cause and cardiovascular mortality—particularly in ACS—as well as major adverse cardiac and cerebrovascular events, at the cost of increased major and minor bleeding [117]. Accordingly, ACS patients undergoing CABG who are not at high bleeding risk should initiate or resume DAPT once hemostasis is secured and continue for 12 months [104]. Ticagrelor or prasugrel should be preferred when not contraindicated, whereas clopidogrel may be used when these agents are unavailable or not tolerated [104]. In patients requiring oral anticoagulation, anticoagulation plus single antiplatelet therapy should be resumed as soon as feasible, and triple therapy should be avoided because of excess bleeding risk [104].

### 6.2. Antithrombotic Therapy in Atrial Fibrillation and Other Indications for Oral Anticoagulation

Atrial fibrillation (AF) frequently coexists with CAD, giving rise to a clinically challenging scenario in which competing antithrombotic strategies are required [118,119]. In patients with atrial fibrillation, long-term oral anticoagulation is essential to prevent ischemic stroke and systemic embolism, whereas in patients with CAD antiplatelet therapy is central to reducing the risk of stent thrombosis. Although prolonged triple antithrombotic therapy (TAT) may appear theoretically advantageous, it is now discouraged because of a markedly increased risk of bleeding, which is associated with adverse clinical outcomes [119].

Current European guideline recommendations on antithrombotic therapy for patients with AF undergoing PCI are based on six randomized trials [120,121,122,123,124,125]. The WOEST trial first challenged the conventional triple-therapy paradigm, showing that oral anticoagulation plus clopidogrel without aspirin significantly reduced bleeding as compared with triple therapy (HR 0.36; CI 95% [0.26–0.50]; *p* < 0.0001), without an apparent increase in thrombotic events, although the study was not powered for ischemic outcomes [120]. Notably, the dual antithrombotic therapy (DAT) group was compared with a prolonged TAT regimen lasting 1 year, which is no longer considered standard of care. Subsequently, the ISAR-TRIPLE trial evaluated two different durations of TAT in patients on oral anticoagulation undergoing PCI, showing that a shorter 6-week regimen was not superior to a 6-month regimen in terms of 9-month net clinical benefit (HR 1.14, 95% CI 0.68–1.91; *p* = 0.64) [121]. However, in a landmark analysis between 6 weeks and 9 months, bleeding was significantly reduced with DAT compared with TAT (HR 0.68, 95% CI 0.47–0.98; *p* = 0.04) [121].

With the introduction of DOACs, several randomized trials further reinforced the concept of antithrombotic de-escalation. PIONEER AF-PCI, RE-DUAL PCI, AUGUSTUS, and ENTRUST-AF PCI consistently showed that DOAC-based dual therapy—combining a DOAC with a P2Y_12_ inhibitor—reduced bleeding compared with vitamin K antagonist (VKA)–based TAT, with broadly similar rates of ischemic events [122,123,124,125]. The PIONEER AF-PCI trial randomized 2124 patients with AF undergoing PCI to three treatment strategies: low-dose rivaroxaban (15 mg once daily) plus a P2Y_12_ inhibitor for 12 months, very-low-dose rivaroxaban (2.5 mg twice daily) plus DAPT for 1, 6, or 12 months, or standard VKA-based TAT [122]. Both rivaroxaban regimens significantly reduced clinically relevant bleeding compared with VKA-based TAT (HR 0.59, 95% CI 0.47–0.76 and HR 0.63, 95% CI 0.50–0.80, respectively), with similar rates of cardiovascular death, myocardial infarction, or stroke [122]. However, despite a lack of power for the assessment of ischaemic outcomes, the risk for stroke and stent thrombosis appeared to be numerically higher with both rivaroxaban-based regimens [122,126]. In RE-DUAL PCI, dabigatran 110 mg twice daily plus a P2Y_12_ inhibitor was superior to TAT in reducing major or clinically relevant non-major bleeding (HR 0.52, 95% CI 0.42–0.63), whereas dabigatran 150 mg twice daily plus a P2Y_12_ inhibitor was non-inferior (HR 0.72, 95% CI 0.58–0.88), with similar ischemic outcomes [123]. Moreover, the pooled dabigatran groups were non-inferior to TAT with respect to the composite endpoint of death, myocardial infarction, stroke, systemic embolism, or unplanned revascularization (HR 1.04, 95% CI 0.84–1.29; *p* = 0.005 for non-inferiority) [123]. In AUGUSTUS, 4614 patients with AF undergoing PCI or medically managed ACS receiving a P2Y_12_ inhibitor were randomized in a 2 × 2 factorial design to apixaban (5 mg twice daily) or VKA and to aspirin or placebo for 6 months [124]. Apixaban reduced major or clinically relevant non-major bleeding (HR 0.69, 95% CI 0.58–0.81) and death or hospitalization (HR 0.83, 95% CI 0.74–0.93) compared with VKA, while aspirin withdrawal further reduced bleeding (HR 1.89, 95% CI 1.59–2.24) without significant differences in ischemic outcomes [124]. However, the effects of aspirin withdrawal should be interpreted cautiously, as patients randomized to placebo also received a short course of TAT (median time from PCI to randomization: 6 days), and numerically higher rates of myocardial infarction and stent thrombosis were observed in the placebo group. Notably, a subsequent subanalysis showed that the benefit of TAT was mainly confined to the early post-PCI period and was outweighed by the risk of severe bleeding after 30 days [127]. Finally, in ENTRUST-AF PCI, patients were randomized to edoxaban 60 mg once daily plus a P2Y_12_ inhibitor for 12 months or VKA-based TAT with aspirin, which was administered for at least 1 month and for up to 12 months at the discretion of the investigator [125]. Edoxaban-based DAT demonstrated non-inferiority, for major or clinically relevant non-major bleeding (HR 0.83, 95% CI 0.65–1.05), with similar efficacy outcomes [125].

Taken together, these trials have consolidated the evidence base supporting simplified antithrombotic regimens that incorporate DOACs and favor early discontinuation of aspirin, thereby improving safety without compromising efficacy in patients with atrial fibrillation undergoing PCI [128].

Accordingly, ESC guidelines recommend in ACS patients triple therapy for up to 1 week (Class I, Level of Evidence A), with extension to a maximum of 1 month in patients with a predominant ischemic risk (Class IIa, Level of Evidence C) [84,129]. Thereafter, dual therapy—preferably with clopidogrel—should be continued for up to 12 months (Class I, Level of Evidence A), with the option of discontinuation at 6 months in selected patients (Class IIb, Level of Evidence B) [84]. In patients with CCS, similar principles apply: triple therapy should be discontinued within 1 week, and dual therapy should be maintained for up to 6 months in patients without high ischemic risk or up to 12 months in those at high ischemic risk [19].

The rationale for oral anticoagulant monotherapy extends beyond AF to other clinical conditions requiring long-term anticoagulation, including mechanical heart valves, venous thromboembolism, mitral stenosis, and left ventricular thrombus. In patients with mechanical heart valves, current ESC valvular heart disease guidelines suggest that the addition of low-dose aspirin (75–100 mg daily) to VKA therapy may be considered in selected individuals with concomitant symptomatic atherosclerotic disease, provided that bleeding risk is low (Class IIa, Level of Evidence B) [130]. In contrast, DAPT is not recommended for the prevention of prosthetic valve thrombosis [130]. For other conditions, such as mitral stenosis and left ventricular thrombus, the evidence supporting the routine addition of antiplatelet therapy to oral anticoagulation remains limited, underscoring the need for further dedicated studies [8].

### 6.3. Antithrombotic Therapy in Elderly

In older patients, CAD is generally more prevalent and anatomically complex, making PCI a frequent revascularization strategy [131]. Although current guidelines recommend 6 or 12 months of DAPT with a P2Y_12_ inhibitor according to clinical presentation, in elderly patients the frequently elevated bleeding risk has prompted consideration of alternative antiplatelet strategies [19,84]. In the POPular AGE trial, among patients ≥ 70 years with NSTE-ACS, clopidogrel plus aspirin for 12 months reduced bleeding compared with ticagrelor, without increasing death, myocardial infarction, stroke, or bleeding [132]. Although more potent P2Y_12_ inhibitors reduce ischemic events in pivotal trials, subgroup analyses in older patients show attenuated ischemic benefit and higher bleeding risk [56,57,133]. Consistently, SWEDEHEART registry data in patients ≥ 80 years with ACS showed similar ischemic event rates with ticagrelor and clopidogrel, but higher mortality and bleeding with ticagrelor [134].

In patients at high bleeding risk, the duration of DAPT after PCI can therefore be substantially shortened. Current ESC guidelines do not endorse a specific antiplatelet strategy tailored exclusively to older patients but acknowledge multiple options for reducing the intensity and duration of antiplatelet therapy [19,84]. A uniform, “one-size-fits-all” approach is not applicable in this heterogeneous population. Instead, optimal management requires clinical expertise to integrate patient-specific characteristics, bleeding and ischemic risk scores, and coronary anatomy in order to individualize antiplatelet regimens and their duration.

### 6.4. Sex Differences

The incidence and mechanisms of coronary thrombosis exhibit sex-related differences, particularly among premenopausal women, in whom cyclical fluctuations in sex hormones modulate platelet reactivity and coagulation pathways [135,136]. In clinical practice, women with ACS tend to present at an older age than men and have a higher prevalence of cardiovascular and systemic comorbidities, including diabetes mellitus, hypertension, and chronic kidney disease [137]. Despite these differences, available evidence indicates that the efficacy of contemporary antithrombotic strategies is broadly similar in women and men with ACS, irrespective of revascularization status [137]. Pooled analyses have not shown a significant interaction between sex and the efficacy of aspirin for secondary prevention [21]. In the setting of DAPT, although women have a higher absolute risk of major ischemic events, meta-analytic data suggest that the relative benefit of clopidogrel added to aspirin may be attenuated compared with that observed in men [138]. By contrast, randomized trials evaluating prasugrel and ticagrelor, have not demonstrated clinically meaningful sex-based differences in efficacy, notwithstanding numerically greater treatment effects for selected endpoints in men [56,57]. Overall, current data do not support sex-specific modulation of antithrombotic efficacy in secondary prevention [137]. However, the persistent underrepresentation of women in randomized trials and their higher rates of treatment discontinuation limit the robustness of safety and efficacy estimates [137]. Accordingly, decisions regarding the selection and duration of antithrombotic therapy after ACS or PCI should be guided primarily by the individual balance of ischemic and bleeding risk and the burden of comorbid conditions, rather than by biologic sex alone [137,139].

### 6.5. Ethnicity

Ethnic differences may influence both thrombotic and bleeding risk, as well as response to antithrombotic therapy. Notably, East Asian patients have been consistently associated with a lower risk of ischemic events and a higher propensity for bleeding compared with Western populations, a phenomenon often referred to as the “East Asian paradox” [140]. These differences have been attributed to variations in platelet reactivity, pharmacogenomics (including CYP2C19 polymorphisms affecting clopidogrel metabolism) and clinical profiles. Despite a higher prevalence of high on-treatment platelet reactivity with clopidogrel, East Asian patients do not derive the same magnitude of ischemic benefit from more potent P2Y_12_ inhibitors, while experiencing excess bleeding. Notably, a large number of trials of post-PCI antithrombotic strategies have been conducted in East Asian populations, generating a solid body of evidence in these subjects and questioning the external validity of such strategies in Westerners [71]. Overall, current evidence does not support ethnicity-based personalization of antithrombotic regimens; instead, patient-specific risk profiles, comorbidities, and pharmacogenetic considerations should be taken into account.

### 6.6. Cancer

In patients with cancer, the management of antithrombotic therapy is challenging owing to the delicate balance between an increased thrombotic risk and a heightened risk of bleeding [141]. Malignancy and its treatments promote a prothrombotic milieu; however, concomitant conditions such as thrombocytopenia, coagulation abnormalities, and overall frailty substantially increase the risk of hemorrhagic complications [141]. As a result, these patients are generally considered to be at high bleeding risk. The occurrence of ACS or CCS in this population is associated with a higher incidence of major cardiovascular events, mortality, and bleeding complications [84].

Despite the clinical importance of this issue, robust evidence remains limited, as patients with active cancer are frequently excluded from randomized clinical trials evaluating antithrombotic therapies in CAD [142]. In patients with cancer presenting with ACS, clopidogrel is generally preferred as the P2Y_12_ inhibitor because of its more favorable bleeding profile compared with more potent agents [81]. DAPT may be administered with careful consideration of platelet count thresholds (e.g., >30,000/µL), whereas prasugrel, ticagrelor, and glycoprotein IIb/IIIa inhibitors are typically avoided in the presence of significant thrombocytopenia [84]. Similarly, in CCS, management parallels that of patients without cancer; however, decisions regarding the type and duration of antithrombotic therapy, as well as revascularization strategies, should take into account life expectancy, coexisting conditions, potential drug–drug interactions with anticancer therapies, and the individual balance between ischemic and bleeding risks [19].

When an indication for oral anticoagulation coexists (e.g., atrial fibrillation or recent venous thromboembolism), combination strategies further increase bleeding risk and should be minimized. Current recommendations from the ESC and the European Hematology Association suggest that, in patients with mild thrombocytopenia (platelet count > 50,000/µL), antithrombotic therapy may generally follow the same principles as in patients without cancer, with appropriate clinical monitoring [142]. In contrast, in patients with moderate-to-severe thrombocytopenia (platelet counts between 25,000 and 50,000/L), combined antiplatelet and anticoagulant therapy is usually avoided, and treatment is often limited to single antiplatelet therapy, most commonly with low-dose aspirin [142]. In the absence of high-quality evidence, treatment decisions should be guided by an assessment of the individual patient’s ischemic and bleeding risks, the underlying oncologic condition, with close coordination among the treating specialists.

## 7. Practical Clinical Implications

Despite dual antiplatelet therapy after PCI and long-term aspirin monotherapy having historically represented the cornerstone of secondary prevention in patients with coronary artery disease, emerging evidence from randomized trials and meta-analyses is progressively reshaping clinical practice toward a more personalized antithrombotic approach. Rather than adopting a uniform strategy, clinicians should tailor antiplatelet therapy according to the dynamic balance between ischemic and bleeding risk, which frequently evolves over time. In the early phase after PCI, when thrombotic risk is highest, standard DAPT remains appropriate for most patients. However, individuals at high bleeding risk—including elderly patients, those with anemia, chronic kidney disease, prior bleeding events, frailty, active cancer, or concomitant need for oral anticoagulation—may benefit from abbreviated DAPT regimens followed by P2Y_12_ inhibitor monotherapy. In these patients, early aspirin withdrawal may reduce bleeding complications without significantly compromising ischemic protection. Conversely, patients with high ischemic risk features, such as prior myocardial infarction, diabetes mellitus, multivessel coronary artery disease, recurrent ischemic events, peripheral artery disease, or complex PCI, may benefit from prolonged or intensified antiplatelet strategies, provided bleeding risk remains acceptable. For example, patients fulfilling PEGASUS-TIMI 54 criteria may be considered for extended therapy with aspirin plus ticagrelor 60 mg twice daily. Importantly, antiplatelet therapy should not be viewed as a static decision made at hospital discharge. New clinical scenarios—including the need for non-cardiac surgery, development of malignancy, recurrent bleeding, new atrial fibrillation requiring anticoagulation, or recurrent ischemic events—should prompt re-evaluation of the antithrombotic regimen. This concept of temporal modulation reflects the progressive decline in ischemic risk after PCI, while bleeding risk often persists or increases over time. Long-term maintenance therapy also represents an evolving area. Recent evidence suggesting superior ischemic protection with similar bleeding risk for P2Y_12_ inhibitor monotherapy compared with aspirin challenges the historical role of aspirin as the default long-term antiplatelet strategy. Ultimately, the optimal antithrombotic strategy after PCI should be dynamic and patient-centered, adapting over time according to clinical presentation, procedural complexity, comorbidities, and the evolving balance between thrombotic and bleeding risk.

## 8. Conclusions

In CAD, antithrombotic therapy remains central across the continuum from primary prevention to long-term secondary prevention, but its intensity and—when two or more agents are combined—its duration should reflect the balance between ischemic protection and bleeding risk. In primary prevention, evidence supports at best a marginal net benefit of aspirin in highly selected individuals, while other compounds remain understudied. After PCI, DAPT is the standard of care, but its optimal duration remains debated and depends on clinical presentation as well as thrombotic and bleeding risk. In long-term maintenance, SAPT remains the standard strategy, with emerging evidence favouring clopidogrel over aspirin, whereas intensified regimens may be considered in patients with persistently high ischemic risk, recurrent events, or comorbidities such as peripheral artery disease. Special settings require dedicated approaches, with anticoagulants playing a role in the procedural setting as parenteral formulations, and in the long-term management of patients with myocardial infarction who also have atrial fibrillation or mechanical heart valves. Ongoing studies are primarily exploring more personalized antithrombotic approaches, especially after PCI, in an effort to improve the overall benefit–risk profile of treatment.

## Figures and Tables

**Table 1:** Trial of short DAPT followed by aspirin monotherapy.

Trial (Year)	Population	Sample Size	Treatment Comparison	Follow-Up	Primary End Point	Results
**EXCELLENT (2012)**	CCS 49%, ACS 51%	1443	6-month DAPT followed by aspirin monotherapy vs. 12-month DAPT	12 mo	Cardiac death, MI, or TVR	4.8% vs. 4.3%; *p* < 0.001 for noninferiority
**PRODIGY (2012)**	CCS 26%, ACS 74%	1970	6-month DAPT followed by aspirin monotherapy vs. 24-month DAPT	24 mo	Death, MI, or stroke	10.0% vs. 10.1%; HR 0.98; 95% CI, 0.74–1.29; *p* = 0.91
**SECURITY (2014)**	CCS 61%, ACS 39%	1399	6-month DAPT followed by aspirin monotherapy vs. 12-month DAPT	12 mo	Cardiac death, MI, stroke, stent thrombosis, or BARC 3–5 bleeding	4.5% vs. 3.7%; 95% CI, −2.4 to 1.7; *p* = 0.47
**ITALIC (2015)**	CCS 76%, ACS 23%	1850	6-month DAPT followed by aspirin monotherapy vs. 24-month DAPT	12–24 mo	Death, MI, TVR, stroke, or TIMI major bleeding	1.5% vs. 1.6%; 95% CI, −1.04 to 1.26; *p* < 0.001 for noninferiority
**ISAR-SAFE (2015)**	CCS 61%, ACS 39%	4000	6-month DAPT followed by aspirin monotherapy vs. 12-month DAPT	12 mo	Death, MI, stent thrombosis, stroke, or TIMI major bleeding	1.5% vs. 1.6%; *p* < 0.001 for noninferiority
**I-LOVE-IT 2 (2016)**	CCS 15%, ACS 85%	1829	6-month DAPT followed by aspirin monotherapy vs. 12-month DAPT	12 mo	Cardiovascular death, target-vessel MI, or clinically indicated TLR	6.8% vs. 5.9%; 95% CI, −1.37 to 3.11; *p* = 0.007 for noninferiority
**NIPPON (2017)**	CCS 68%, ACS 32%	3773	6-month DAPT followed by aspirin monotherapy vs. 18-month DAPT	18 mo	Death, MI, stroke, or major bleeding	2.1% vs. 1.5%; 95% CI, −1.5 to 0.3
**OPTIMA-C (2018)**	CCS 49%, ACS 51%	1368	6-month DAPT followed by aspirin monotherapy vs. 12-month DAPT	12 mo	Cardiac death, MI, or TLR	1.2% vs. 0.6%; 95% CI, −0.4 to 1.6; *p* < 0.05 for noninferiority
**RESET (2012)**	CCS 46%, ACS 54%	2117	3-month DAPT followed by aspirin monotherapy vs. 12-month DAPT	12 mo	Cardiovascular death, MI, stent thrombosis, TVR, or bleeding	4.7% vs. 4.7%; 95% CI, −2.5 to 2.5; *p* < 0.001 for noninferiority
**OPTIMIZE (2013)**	CCS 68%, ACS 32%	3119	3-month DAPT followed by aspirin monotherapy vs. 12-month DAPT	12 mo	Death, MI, stroke, or major bleeding	6.0% vs. 5.8%; 95% CI, −1.52 to 1.86; *p* = 0.002 for noninferiority
**One-Month DAPT (2021)**	CCS 61%, ACS 39%	3020	1-month DAPT vs. 6–12-month DAPT	12 mo	Cardiac death, MI, stroke, TVR, or major bleeding	5.9% vs. 6.5%; upper 97.5% CI, 1.33; *p* < 0.001 for noninferiority
MASTER DAPT (2021) *	CCS 51%, ACS 49%	4579	1-month abbreviated DAPT vs. standard DAPT (≥3–6 months)	12 mo	NACE; MACE; BARC 2–5 bleeding	NACE: 7.5% vs. 7.7% 95% CI, −1.80 to 1.33; *p* < 0.001 for noninferiority MACE: 6.1% vs. 5.9%; *p* = 0.001 for noninferiority BARC 2–5 bleeding: 6.5% vs. 9.4%; *p* < 0.001 for superiority

**Abbreviations:** ACS, acute coronary syndrome; BARC, Bleeding Academic Research Consortium; CCS, chronic coronary syndrome; CI, confidence interval; DAPT, dual antiplatelet therapy; HR, hazard ratio; MACE, major adverse cardiovascular events; MI, myocardial infarction; mo, months; NACE, net adverse clinical events; TIMI, Thrombolysis in Myocardial Infarction; TLR, target-lesion revascularization; TVR, target-vessel revascularization. * After discontinuation of DAPT, clopidogrel was used as monotherapy in approximately 54% of patients.

**Table 2:** Trial of short DAPT followed by P2Y_12_ receptor inhibitor monotherapy.

Trial (Year)	Population	Sample Size	Comparison	Follow-Up	Primary Endpoint	Results
**GLOBAL LEADERS** **(2018)**	CCS 53%, ACS 47%	15,968	One-month DAPT followed by ticagrelor monotherapy for 23 months vs. DAPT with ticagrelor (ACS) or clopidogrel (CCS) for 12 months followed by aspirin monotherapy	2 years	Death or MI	3.8% vs. 4.4%; Rate ratio 0.87; 95% CI 0.75 to 1.01; *p* = 0.073
**SMART-CHOICE** **(2019)**	CCS 42%, ACS 58%	2993	Three-month DAPT followed by P2Y_12_ inhibitor monotherapy vs. 12-month DAPT	1 year	Death, MI, or stroke	2.9% vs. 2.4%; Difference 0.4%; one-sided 95% CI 1.3%; *p* = 0.007 for NI
**STOPDAPT-2** **(2019)**	CCS 62%, ACS 38%	3045	One-month DAPT followed by clopidogrel monotherapy vs. 12-month DAPT	1 year	CV death, MI, stroke, ST, or TIMI major or minor bleeding	2.4% vs. 3.7%; Difference −1.34%; 95% CI −2.57% to −0.11%; *p* < 0.001 for NI; *p* = 0.04 for superiority
**TWILIGHT** **(2019)**	CCS 36%, ACS 64%	7119	Three-month DAPT followed by ticagrelor monotherapy vs. 15-month DAPT	15 months	BARC 2, 3 or 5 bleeding	4.0% vs. 7.1%; HR 0.56; 95% CI 0.45 to 0.68; *p* < 0.001
**SHARE** **(2024)**	CCS 26%, ACS 74%	1387	Three-month DAPT followed by P2Y_12_ inhibitor monotherapy vs. 12-month DAPT	1 year	Composite of cardiac death, MI, ST, stroke, ischemia-driven target lesion revascularization, or BARC 3–5 bleeding	1.7% vs. 2.6%; Difference −0.93%; 95% CI −2.64 to 0.77; *p* < 0.001 for NI

**Abbreviations:** ACS, acute coronary syndrome; BARC, Bleeding Academic Research Consortium; CCS, chronic coronary syndrome; CI, confidence interval; CV, cardiovascular; DAPT, dual antiplatelet therapy; HR, hazard ratio; MI, myocardial infarction; NI, noninferiority; ST, stent thrombosis.

**Table 3:** Ongoing randomized clinical trials on DAPT strategies in CCS patients.

Trial Name (Clinicaltrials.gov ID)	Sample Size	Population	Interventional Strategy	Control Strategy	Primary Endpoint
**TAILOR-DAPT** **(NCT05732701)**	2788	CCS or ACS	DAPT discontinuation guided by risk stratification	Standard DAPT	NACE at 1 year
**OPTIMIZE-APT** **(NCT05418556)**	3994	CCS or ACS	1-month DAPT (aspirin plus clopidogrel) followed by 11-month clopidogrel alone for CCS; 3-month DAPT (aspirin plus ticagrelor or prasugrel) followed by 9-month P2Y_12_ inhibitor monotherapy for ACS	12-month DAPT	(1) BARC 2, 3, or 5 at 1 year (2) MACE at 1 year (3) NACE at 1 year
**ANGIODAPT** **(NCT05952206)**	2312	CCS or ACS	1-month DAPT followed by P2Y_12_ inhibitor monotherapy	6- or 12-month DAPT (according to clinical presentation)	BARC 2, 3, or 5 at 1 year
**SHORTDAPT (NCT06648720)**	3566	CCS or ACS	1-month DAPT followed by P2Y_12_ inhibitor monotherapy	12-month DAPT	NACCE at 1 year
**GENOSS-DAPT (NCT05770674)**	2186	CCS or ACS (no MI)	1-month DAPT followed by clopidogrel monotherapy	12-month DAPT	NACE at 1 year
**TICALONE (NCT06509893)**	5400	CCS	Ticagrelor (90 mg bid) monotherapy	6-month DAPT	MACE at 6 months
**PROMOTE (NCT06916520)**	300	CCS or ACS	Prasugrel 5 mg monotherapy	12-month DAPT	NACE at 1 year

**Abbreviations:** ACS, acute coronary syndrome; BARC, Bleeding Academic Research Consortium; bid, twice daily; CCS, chronic coronary syndrome; DAPT, dual antiplatelet therapy; MACE, major adverse cardiovascular events; MI, myocardial infarction; NACE, net adverse clinical events; NACCE, net adverse cardiovascular and cerebrovascular events.

**Table 4:** Trial of short DAPT by discontinuation of P2Y_12_ receptor inhibitor.

Trial (Year)	Population	Sample Size	Treatment Comparison	Follow-Up	Primary End Point	Results
**DAPT-STEMI** **(2018)**	STEMI event-free at 6 months after PCI	1100	Six-month DAPT followed by aspirin monotherapy vs. 12-month DAPT	18 mo	All-cause death, MI, any revascularization, stroke, or TIMI major bleeding	4.8% vs. 6.6%; HR 0.73; 95% CI 0.41 to 1.27; *p* = 0.26
**SMART-DATE** **(2018)**	ACS	2712	Six-month DAPT followed by aspirin monotherapy vs. 12-month DAPT	12 mo	All-cause death, MI, or stroke	4.7% vs. 4.2%; Difference 0.5%; upper one-sided 95% CI 1.8%; *p* = 0.03 for NI
**REDUCE** **(2019)**	ACS	1496	Three-month DAPT followed by aspirin monotherapy vs. 12-month DAPT	12 mo	All-cause death, MI, ST, stroke, target vessel revascularization, or BARC 2, 3 or 5 bleeding	8.2% vs. 8.4%HR 0.97; 95% CI 0.68 to 1.39; *p* < 0.001 for NI

**Abbreviations:** ACS, acute coronary syndrome; BARC, Bleeding Academic Research Consortium; CI, confidence interval; DAPT, dual antiplatelet therapy; HR, hazard ratio; MI, myocardial infarction; NI, non-inferiority; PCI, percutaneous coronary intervention; ST, stent thrombosis; STEMI, ST-segment elevation myocardial infarction; TIMI, Thrombolysis in Myocardial Infarction.

**Table 5:** Trial of short-DAPT by discontinuation of ASA.

Trial (Year)	Population	Sample Size	Treatment Comparison	Follow-Up	Primary End Point	Results
**TICO** **(2020)**	ACS	3056	Three-month DAPT followed by ticagrelor monotherapy vs. 12-month DAPT	12 mo	TIMI major bleeding, all-cause death, MI, ST, stroke, or TVR	3.9% vs. 5.9%; HR 0.66; 95% CI 0.48 to 0.92; *p* = 0.01
**STOPDAPT-2 ACS** **(2022)**	ACS	4169	One-month DAPT followed by clopidogrel monotherapy vs. 12-month DAPT	12 mo	CV death, MI, stroke, ST, or TIMI major or minor bleeding	3.2% vs. 2.8%; 95% CI −0.68% to 1.42%; *p* = 0.06 for NI
**STOPDAPT-3** **(2023)**	ACS or HBR (either CCS or ACS)	5966	Prasugrel (3.75 mg/day) monotherapy vs. one-month DAPT with aspirin and prasugrel (3.75 mg/day)	1 month	(1) BARC 3 or 5 bleeding (2) CV death, MI, definite ST, or ischemic stroke	(1) 4.5% vs. 4.7%; HR 0.95; 95% CI 0.75 to 1.20; *p* = 0.66 for superiority (2) 4.1% vs. 3.7%; HR 1.12; 95% CI 0.87 to 1.45; *p* = 0.001 for NI
**T-PASS** **(2024)**	ACS	2850	Ticagrelor monotherapy after <1 month of DAPT vs. 12-month DAPT	12 mo	All-cause death, MI, definite or probable ST, stroke, or BARC 3–5 bleeding	2.8% vs. 5.2%; HR 0.54; 95% CI 0.37 to 0.80; *p* < 0.001
**ULTIMATE-DAPT** **(2024)**	ACS	3400	One-month DAPT followed by ticagrelor monotherapy vs. 12-month DAPT	12 mo	(1) BARC 2–5 bleeding (2) CV death, MI, ischemic stroke, definite ST, or clinically driven TVR	(1) 2.1% vs. 4.6%; HR 0.45; 95% CI 0.30 to 0.66; *p* < 0.0001 (2) 3.6% vs. 3.7%; 95% CI −1.4% to 1–2%; *p* < 0.0001 for NI
**4D-ACS** **(2025)**	ACS	656	One-month DAPT with prasugrel 10 mg followed by prasugrel 5 mg monotherapy vs. 12-month DAPT with aspirin and prasugrel 5 mg	12 mo	All-cause death, MI, stroke, ischemia-driven TVR, or BARC 2, 3 or 5 bleeding	4.9% vs. 8.8%; 95% CI −6.7% to −0.2%; *p* = 0.014 for NI
**NEOMINDSET** **(2025)**	ACS	3410	Periprocedural DAPT followed by ticagrelor or prasugrel monotherapy vs. 12-month DAPT	12 mo	(1) All-cause death, MI, stroke, or urgent TVR (2) BARC 2, 3 or 5 bleeding	(1) 7.0% vs. 5.5%; 95% CI −0.16 to 3.10; *p* = 0.11 for NI (2) 2.0% vs. 4.9%; HR 0.40; 95% CI 0.26 to 0.59
**TARGET-FIRST** **(2025)**	MI	2246	One-month DAPT followed by P2Y_12_ inhibitor monotherapy vs. 12-month DAPT	11 mo	All-cause death, MI, ST, stroke, or BARC 3 or 5 bleeding	2.1% vs. 2.2%; 95% CI −1.39 to 1.20; *p* = 0.02 for NI

**Abbreviation:** ACS, acute coronary syndrome; BARC, Bleeding Academic Research Consortium; CCS, chronic coronary syndrome; CI, confidence interval; CV, cardiovascular; DAPT, dual antiplatelet therapy; HBR, high bleeding risk; HR, hazard ratio; MI, myocardial infarction; mo, months; NI, non-inferiority; ST, stent thrombosis; TIMI, Thrombolysis in Myocardial Infarction; TVR, target vessel revascularization.

**Table 6:** Ongoing randomized clinical trials on DAPT strategies in ACS patients.

Trial Name (Clinicaltrials.gov ID)	Sample Size	Population	Interventional Strategy	Control Strategy	Primary Endpoint
**PREMIUM** (NCT05709626)	2258	STEMI	Prasugrel monotherapy for 12 months	Twelve-month DAPT with prasugrel	MACE at 1 year
**STARS-DAPT** (NCT05785897)	350	STEMI	Antiplatelet monotherapy with prasugrel or ticagrelor	Twelve-month DAPT	(1) MACCE at 1 year (2) BARC 3 or 5 at 1 year
**COMPARE STEMI-ONE** (NCT05491200)	1656	STEMI	Prasugrel-based short DAPT (30–45 days) followed by prasugrel monotherapy	Twelve-month DAPT with prasugrel	NACE at 11 months
**SORT OUT XII DAPT Duration Trial** (NCT06718179)	3150	ACS patients undergoing PCI	One-month DAPT followed by prasugrel monotherapy for 11 months	Twelve-month DAPT with prasugrel	(1) BARC 2–5 bleeding at 1 year (2) MACCE at 1 year
**TIGER** (NCT04255602)	2120	ACS patients undergoing PCI	One-week DAPT with ticagrelor 90 mg bid followed by DAPT with ticagrelor 60 mg bid for 1 year	Twelve-month DAPT with ticagrelor 90 mg bid	NACE at 1 year
**MATE** (NCT04937699)	2690	ACS patients undergoing PCI	One-month DAPT with ticagrelor followed by ticagrelor monotherapy for 5 months, followed by clopidogrel monotherapy for 6 months	Twelve-month DAPT with ticagrelor	NACE from 1 to 12 months after PCI
**ELECTRA-SIRIO** (NCT04718025)	4500	ACS patients undergoing PCI	60 mg ticagrelor plus aspirin or 60 mg ticagrelor plus placebo	Twelve-month DAPT with ticagrelor 90 mg bid	(1) Death from any cause, nonfatal MI, or nonfatal stroke (2) BARC 2–5 bleeding at 1 year

**Abbreviations:** ACS, acute coronary syndrome; BARC, Bleeding Academic Research Consortium; bid, twice daily; DAPT, dual antiplatelet therapy; MACCE, major adverse cardiovascular and cerebrovascular events; MACE, major adverse cardiovascular events; MI, myocardial infarction; NACE, net adverse clinical events; PCI, percutaneous coronary intervention; STEMI, ST-segment–elevation myocardial infarction.

**Table 7:** Trial of long-term antiplatelet therapy.

Trial (Year)	Population	Sample Size	Comparison	Follow-Up	Primary Endpoint	Results
**CHARISMA (2006)**	Clinically evident cardiovascular disease or multiple risk factors	15,603	Clopidogrel vs. placebo on top of aspirin	28 mo.	Efficacy: CV death, MI, or stroke Safety: GUSTO severe bleeding	Efficacy: 6.8% vs. 7.3%; *p* = 0.22 Safety: 1.7% vs. 1.3%; *p* = 0.09
**DES LATE (2010)**	CCS 37%, ACS 63%	2701	24 vs. 12 months of DAPT	24 mo.	CV death or MI	1.8% vs. 1.2%; *p* = 0.17
**DAPT** **(2014)**	CCS 54%, ACS 46%	9961	30 vs. 12 months of DAPT with clopidogrel or prasugrel	18 mo.	Efficacy: (1) stent thrombosis; (2) death, MI, or stroke Safety: GUSTO moderate or severe bleeding	Efficacy-stent thrombosis: *p* < 0.001 Efficacy-composite endpoint: *p* < 0.001 Safety: *p* = 0.001
**PEGASUS–TIMI 54** **(2015)**	CCS 100%	21,162	Ticagrelor 60 mg bid vs. ticagrelor 90 mg bid vs. placebo on top of aspirin	36 mo.	Efficacy: CV death, MI, or stroke Safety: TIMI major bleeding	Efficacy-ticagrelor 90 mg: *p* = 0.008 Efficacy-ticagrelor 60 mg: *p* = 0.004 Safety-ticagrelor 90 mg: *p* < 0.001 Safety-ticagrelor 60 mg: *p* < 0.001
**OPTIDUAL** **(2016)**	CCS 65%, ACS 35%	1385	48 vs. 12 months of DAPT	33 mo.	Death, MI, stroke or major bleeding	5.8% vs. 7.5%; *p* = 0.17
**COMPASS (2017)**	CCS 90.6%	27,395	Rivaroxaban (2.5 mg bid) plus aspirin vs. rivaroxaban (5 mg bid) vs. aspirin	23 mo.	CV death, MI, or stroke	Rivaroxaban 2.5 mg + ASA vs. ASA: 4.1% vs. 5.4%; *p* < 0.001 Rivaroxaban 5 mg vs. ASA: 4.9% vs. 5.4%; *p* = 0.12
**THEMIS (2019)**	CCS 100%	19,220	Ticagrelor vs. placebo on top of aspirin	40 mo.	Efficacy: CV death, MI, or stroke Safety: TIMI major bleeding	Efficacy: *p* = 0.04 Safety: *p* < 0.001

**Abbreviations:** ACS, acute coronary syndrome; ASA, acetylsalicylic acid (aspirin); bid, twice daily; CCS, chronic coronary syndrome; CV, cardiovascular; DAPT, dual antiplatelet therapy; GUSTO, Global Utilization of Streptokinase and Tissue Plasminogen Activator for Occluded Coronary Arteries; MI, myocardial infarction; TIMI, Thrombolysis in Myocardial Infarction.

**Table 8:** Ongoing randomized clinical trials on DAPT strategies for long-term antiplatelet therapy.

Trial Name (Clinicaltrials.gov ID)	Sample Size	Population	Interventional Strategy	Control Strategy	Primary Endpoint
**C-MODE** **(NCT05320926)**	3744	CCS or ACS (no MI)	1–3 months DAPT followed by clopidogrel monotherapy	1–3 months DAPT followed by aspirin monotherapy	NACE at 1 year
**A-CLOSE (NCT03947229)**	3200	CCS or ACS at high ischemic or bleeding risk, event-free 12 months post-PCI	Clopidogrel monotherapy	Extended DAPT	NACE at 2 years
**DAPT-MVD (NCT04624854)**	8250	CCS or ACS patients with MVD	Extended DAPT for 12 months after randomization	Aspirin monotherapy	MACCE at 34.3 months

**Abbreviations:** ACS, acute coronary syndrome; CCS, chronic coronary syndrome; DAPT, dual antiplatelet therapy; MACCE, major adverse cardiovascular and cerebrovascular events; MI, myocardial infarction; MVD, multivessel disease; NACE, net adverse clinical events; PCI, percutaneous coronary intervention.

## Data Availability

No new data were generated or analyzed in support of this research.

## Supplementary Materials

The following supporting information can be downloaded at: https://www.mdpi.com/article/10.3390/jcm15114248/s1. A literature search was conducted in PubMed to identify relevant studies on antithrombotic therapy in coronary artery disease published up to 12/03/2026. The following search strategy was used in PubMed: (“Acute Coronary Syndrome” [Mesh] OR ACS OR STEMI OR NSTEMI OR unstable angina OR “Chronic Coronary Syndrome” OR CCS OR stable coronary disease OR chronic coronary disease OR “Coronary Artery Disease” [Mesh]) AND (PCI OR “Percutaneous Coronary Intervention” [Mesh] OR stent* OR “drug-eluting stent*” OR DES) AND (“dual antiplatelet therapy” OR DAPT OR aspirin OR clopidogrel OR prasugrel OR ticagrelor OR P2Y12) AND (duration OR short* OR abbreviated OR de-escalation OR discontinu* OR monotherapy OR aspirin withdrawal) AND (randomized controlled trial[pt] OR random*) NOT (animal[mh] NOT human[mh]).
