# Supplementary material for: Antithrombotic Therapy in Primary and Secondary Prevention of Coronary Artery Disease
Source: J Clin Med. 2026 May 30;15(11):4248. doi: 10.3390/jcm15114248 (PMC13257674; doi:10.3390/jcm15114248)
Supplement: Supplementary file 1 [file jcm-15-04248-s001.zip › jcm-4269075-supplementary.pdf]

Supplementary Material

Research Metodology:

A literature search was conducted in PubMed to identify relevant studies on antithrombotic therapy in coronary artery disease published up to 12/03/2026.

The following search strategy was used in PubMed:

("Acute Coronary Syndrome"[Mesh] OR ACS OR STEMI OR NSTEMI OR unstable angina OR "Chronic Coronary Syndrome" OR CCS OR stable coronary disease OR chronic coronary disease OR "Coronary Artery Disease"[Mesh])
AND (PCI OR "Percutaneous Coronary Intervention"[Mesh] OR stent* OR "drug-eluting stent*" OR DES) AND ("dual antiplatelet therapy" OR DAPT OR aspirin OR clopidogrel OR prasugrel OR ticagrelor OR P2Y12) AND (duration OR short* OR abbreviated OR de-escalation OR discontinu* OR monotherapy OR aspirin withdrawal) AND (randomized controlled trial[pt] OR random*) NOT (animal[mh] NOT human[mh])
